# Mercury Speciation in Hair of Children in Three Communities of the Amazon, Brazil

**DOI:** 10.1155/2014/945963

**Published:** 2014-03-11

**Authors:** Jamile Salim Marinho, Marcelo Oliveira Lima, Elisabeth Conceição de Oliveira Santos, Iracina Maura de Jesus, Maria da Conceição N. Pinheiro, Cláudio Nahum Alves, Regina Celi Sarkis Muller

**Affiliations:** ^1^Evandro Chagas Institute, 316 Km, 07 Road, Levilândia, 67030-000 Ananindeua, PA, Brazil; ^2^Federal University of Pará, 01 Augusto Corrêa Street, Guamá, 66075-110 Belém, PA, Brazil

## Abstract

Children from riverside communities located downstream of gold mining areas may be chronically exposed to relatively high levels of MeHg through the consumption of fish of this region. The objective of this study was to evaluate and compare levels of THg and MeHg in hair of children less than 12 years in communities near mines in the municipality of Itaituba and in communities far from prospecting areas in the city of Abaetetuba. The communities of Itaituba (Barreiras and São Luiz do Tapajós) had THg mean levels of 5.64 ± 5.55 **μ**g*·*g^−1^ (0.43–27.82) and 11.41 ± 7.16 **μ**g.g^−1^ (1.08–28.17), respectively, and an average count of MeHg relative to THg of 92.20% and 90.27%, respectively. In the Maranhão community, the THg average concentrations results were 2.27 ± 2.11 **μ**g*·*g^−1^ (0.13–9.54) and the average values were 93.17% for MeHg. Children of Itaituba had average levels of mercury above the limit established by the World Health Organization (10 **μ**g*·*g^−1^) and the strong correlation coefficient between the communities (*R* = 0.968 and *P* = 0.0001) suggests the hair as an excellent biomarker of human exposure to organic mercury in riverside populations of the Tapajós, which has the intake of fish daily as main source of protein dietary.

## 1. Introduction

In the Amazon, there are records that the industrial and artisanal mining of gold and, possibly, other natural and anthropogenic sources, have increased the concentration of mercury (Hg) in the environment, resulting, through the trophic chain, in process of bioaccumulation in fish consumed by riverside communities. Previous studies related elevated levels of Hg in fish consumption in these communities [1–5].

The mercury is toxic in its various physical and chemical forms, being identified as one of the most dangerous pollutants in the environment [6–8]. This toxicity is amplified in the organomercury forms, particularly methylmercury (MeHg), a substance of high volatility and easily absorbed across biological membranes, with records of efficient bioaccumulation and long-term attachment to the tissues [9, 10].

Previous studies indicate that the bioaccumulation of Hg is harmful to human being, with increments of risk in vulnerable groups such as that, for example, children can receive burden of Hg from mothers infected during pregnancy [11]. By the experimental studies, there are reports on the harmful effects of Hg to the development of human biological systems, with evidence of alterations on the cardiovascular, neurological, immune, and respiratory systems; effects that may persist for entire life [12, 13].

Children are more vulnerable to mercury exposure [14]. Therefore, children from riverside communities located downstream from gold mining areas, may be chronically exposed to relatively high levels of MeHg by fish consumption [15]. From previous studies, it is estimated that MeHg represents approximately 70–90% of the total mercury (THg) consumed by the fish [15–17]. Thus, in these communities, the environmental monitoring of THg and MeHg levels in hair associated with the epidemiological information represents important steps to ensure the quality of life of these populations.

The objective of this study was to perform a quantitative comparison of THg and MeHg levels in children younger than 12 years on populations in near and distant prospecting areas of the Amazon, associating these results with epidemiological data obtained through interviews.

## 2. Material and Methods

### 2.1. Study Area

**Figure 1:**
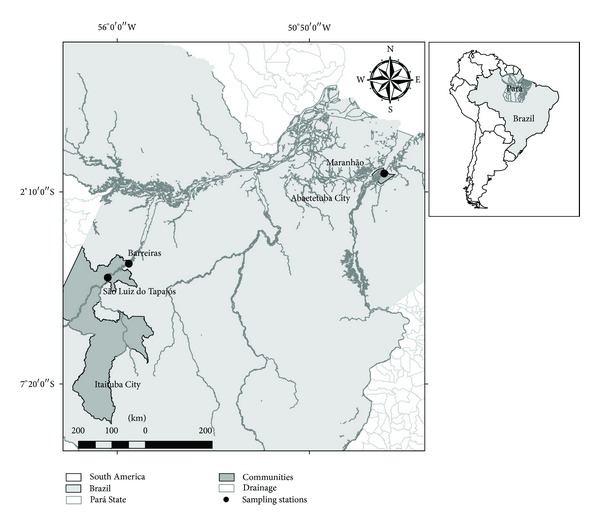
Study area encompassing the communities of Barreiras, São Luiz do Tapajós, and Maranhão.

For this study, children were selected in three Amazonian communities: São Luiz do Tapajós, Barreiras, and Maranhão (Figure 1). The localities of São Luiz do Tapajós (Lat (S): 4°28′11′′ and Long (W): 56°15′20′′) and Barreiras (Lat (S): 4°5′34′′ and Long (W): 55°41′11′′), with 460 and 740 people, respectively, are located on the banks of the Tapajós River, near the town of Itaituba in southwestern Pará. In this region, considered as the Hg exposure, there are records of more than four decades of gold mining [18]. The Maranhão community (Lat (S): 1°39′56′′ and Long (W): 48°49′10′′), with approximately 500 inhabitants, is located on the banks of the Guajará of Beja, a tributary of the right bank of the Pará River, in the northeastern region of the State of Pará, an area where there are no records of exposure to Hg.

### 2.2. Epidemiology

Individuals who reside in the communities selected for this study have similar lifestyles, highlighting the continuous intake of fish as the main protein source. However, these populations are differentiated based on the influences exerted by gold mining activities whose environmental exposure to Hg is higher in those communities located on the banks of the Tapajós River.

The recruitment of individuals was performed by invitation in the communities. The research project was explained to the people responsible for these children, who gave permission by signing the Free and Clarified Consent Term (FCCT). Between 2007 and 2009, a total of 195 children aged 0–12 years old were selected to participate in this study. Of this group, 37 children are residents of the community of Barreiras, 40 are from São Luiz do Tapajós, and 118 are from the Maranhão community.

Epidemiological data, such as age, sex, and length of stay at the place, were obtained through interviews with parental or legal guardian monitoring. Approval for the study procedures was obtained by the Ethics Committee in Research of the Evandro Chagas Institute, by registration number 0013/2008, approved on 09/06/2009.

### 2.3. Sampling and Sample Management

The hair samples were collected from the head occipital area, approximately one (1) cm from the scalp of children with stainless steel dissecting scissors, and stored in paper envelopes properly identified. These samples were then sent to the Division of Mercury in the Laboratory of Toxicology, Section of Environment (SAMAM) of the Evandro Chagas Institute (ECI), located in the town of Ananindeua, State of Pará, Brazil. In the laboratory, the samples were washed with neutral detergent solution (Extran-Merck MA 0-alkaline) (diluted 100 times) to eliminate possible exogenous contamination, deionized water (Milli-Q) to remove sediments, and acetone (Merck) to enable water evaporation, reducing the humidification. After the washing step and subsequent drying, the samples were transferred to 20 mL amber vials and perforated with stainless steel dissecting scissors for greater homogenization [19].

### 2.4. Total Mercury (THg)

In analysis of THg in hair, the methodology developed by [19] was applied that involves the steps of wet digestion, reduction with stannous chloride (SnCl_2_), and quantification by cold vapor atomic absorption spectrometry (CVAAS). In this method, about 0.01 g of each sample was weighed in a 50 mL volumetric flask (Pyrex), and 1 mL of deionized water was added plus 2 mL of HNO_3_ + HClO_4_ (1 : 1) and 5 mL of H_2_SO_4_ and heated for 30 minutes in a hot plate at a temperature of 230°C. After cooling, the solution was measured with deionized water obtained by the water purification system (Millipore, Academic Milli-Q model) to a final volume of 50 mL.

After digestion, 5 mL of sample was added in the reactor vial of the Hg analyzer, Automatic Mercury Analyzer Hg-201 (Sanso Company), with the addition of 1 mL of stannous chloride (SnCl_2_-10%) to reduce Hg^2+^ to Hg^0^. Then, the Hg vapor generated is conducted into a flask containing NaOH at 5 N, that neutralizes the acid vapors, and a flask containing an ice bath, which condenses water vapor. Thereupon, the Hg vapors are transported to the photoabsorption cell to measure absorbance of 253.7 mm. The reading is performed within one minute and can be measured until 0.1 ng of Hg with high accuracy [19].

In the analysis of THg, calibration curves were used: 0, 20, 50, and 100 ng of Hg prepared starting from standard solution of 0.1 ppm MeHg-cysteine.

### 2.5. Methylmercury (MeHg)

In the analysis of MeHg in hair, the simplified method proposed by Akagi [19] was applied, which involves leaching with hydrochloric acid, extraction with toluene, and quantification by gas chromatography with electron capture detector (GC-ECD). In this method, approximately 0.02 g of each sample was weighed and transferred to a round bottom centrifuge tube of 10 mL. Subsequently, 2 drops of ethanol and a small amount of cotton to moisten and cover the sample were added, respectively. For leaching from MeHg, samples were immersed in 3 mL of HCl at 2 N and heated at 100°C for 5 minutes. After cooling, the samples were centrifuged at 1200 rpm for 3 minutes. Then, 1 mL of the supernatant was transferred to a conical tube centrifuge of 10 mL and 2 mL of toluene was added with stirring for 3 minutes to extract the MeHg in the phase of HCl to the phase of toluene. After digestion, 2 *μ*L of sample was injected into the GC-ECD (Yanaco), series G-6800. Glass column of 3.0 mm × 0.75 m was used and packed with 10% KOCl-Hg on Chromosorb W (AW-DMCS, 60-80, Ltd., Kyoto, Japan).

### 2.6. Quality Control

As quality criteria for the analysis of THg and MeHg, Certified Reference Material (CRM) IAEA 86 and IAEA 085 were used.

### 2.7. Statistics

For statistical analyses, the data were submitted to the program Microsoft Excel 2007 and MINITAB 14 software [20]. For results with statistical significance, *P* ≤ 0.05 was used. In correlation analyses, *R* = 1 for perfect correlation; *R* next to ±0.9, for strong correlation; *R* next to ±0.1, for weak correlation; and *R* = 0, for zero correlation were considered [21].

## 3. Results and Discussion

### 3.1. Quality Control

The average results of the analysis of THg and MeHg in the samples from CRM and their reference values are shown in Table 1. The great analytical recovery for THg (99.21%) and MeHg (94.35%) showed that the methodology was effective.

### 3.2. Epidemiology

From the epidemiological information, the children were grouped into different age groups according to the stages of life [18]: (A) breast-feeding (0-1 years), (B) prekindergarten or preschool (2–6 years), and (C) childhood or school (7–12 years). In the three communities, despite the different sample sizes, a similar pattern of distribution of individuals between stages of life evaluated was observed; in other words, in the research, there was the prevalence of children at school phase. Regarding gender, in the Barreiras, the male children (54%) are of larger numbers than the females (46%). On the other hand, in São Luiz do Tapajós, this predominance was higher in female children (60%) than male ones (40%). This inversion was also observed in Maranhão community, in which the number of female children (52.5%) was also higher than male children (47.5%).

Children from Barreiras and São Luiz do Tapajós were also divided into another two groups, in relation to paternal occupation, in order to analyze the importance of familial influences on exposure to Hg: (y) children whose fathers were fisherman and (x) children whose fathers were not fisherman. In Barreiras, the children (x) are of higher amounts (60%) than the children (y) (40%). In contrast, in São Luiz do Tapajós, this profile was higher in children (y) (55%) than in (x) (45%). As regards the duration of breast-feeding, in Barreiras, the average time was about 6 months, while, in São Luiz do Tapajós, this mean increased to 10 months.

### 3.3. Results of HG

The average results of THg and MeHg, standard deviation, and the mean percentage of MeHg in children of the communities investigated are presented in Table 2. The highest average levels of THg (11.41 *μ*g·g^−1^) and MeHg (10.30 *μ*g·g^−1^) in hair of children were found in the community of São Luiz do Tapajós. These values are twice the average of THg (5.64 *μ*g·g^−1^) and MeHg (5.20 *μ*g·g^−1^) found in Barreiras and four times higher than the results of THg (2.27 *μ*g·g^−1^) and MeHg (2.11 *μ*g·g^−1^) in the Maranhão community. These results confirm previous comparative studies with riverside populations of areas exposed and not exposed to Hg, which detect higher levels of THg in the community of São Luiz do Tapajós [15, 18, 22, 23], possibly because of higher proximity of the community with the gold mining area of the Tapajos River Basin [5].

**Figure 2:**
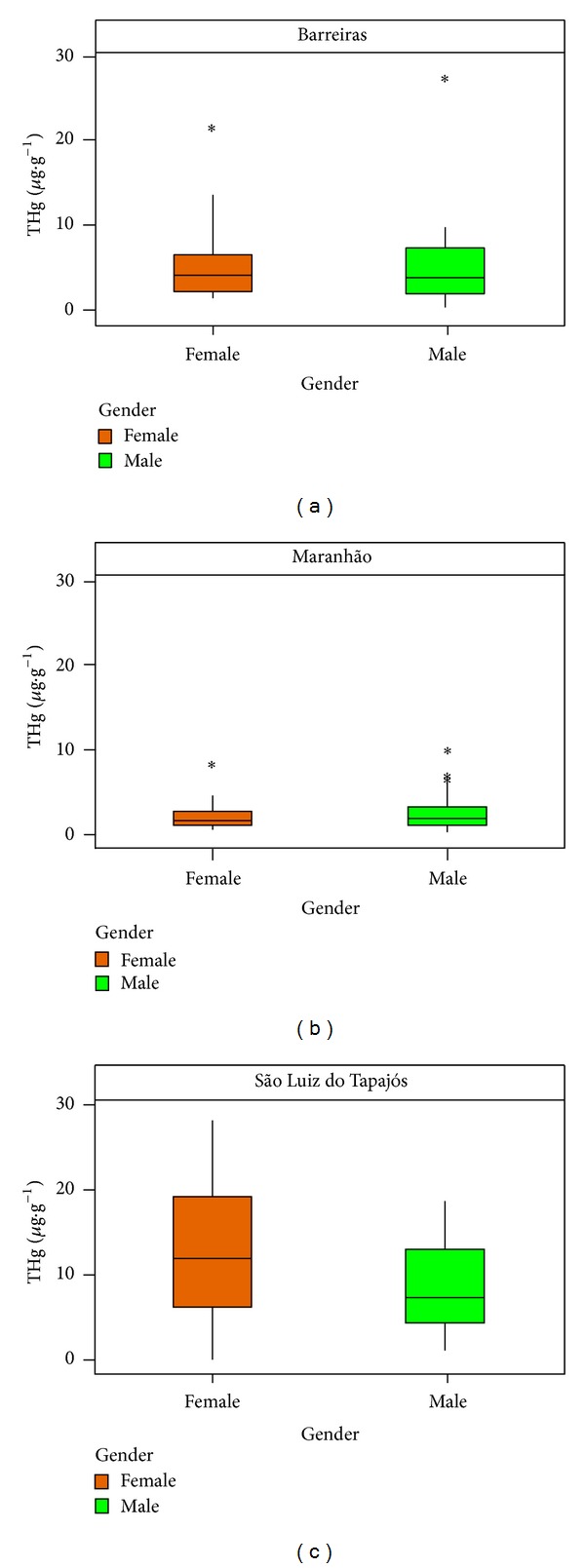
Box plot average levels of THg by gender.

Regarding gender, in the communities of Barreiras and São Luiz do Tapajós, the female children had average levels of THg higher than 4.27 *μ*g·g^−1^ (2.29–4.60) and 5.12 *μ*g·g^−1^ (6.12–19.41), relatively higher than the levels found in male children, which values were 4.1 *μ*g·g^−1^ (2.12–7.61) and 7.73 *μ*g·g^−1^ (4.3–13.2), respectively. In the Maranhão community, the THg highest mean levels were found in males, 1.78 *μ*g·g^−1^ (1.04–2.13), compared to THg levels found in females, 1.50 *μ*g·g^−1^ (1.01–2.61). THg mean levels by gender are shown in Figure 2. In contrast, studies have shown higher levels of mercury in male children from the communities of Barreiras and São Luiz dos Tapajós [18]. Also, the occurrence of outliers in the community of Barreiras is noteworthy, where there was registration of two children; both are sons of fisherman father, with levels of 21.96 *μ*g·g^−1^ (female) and 27.82 *μ*g·g^−1^ (male). In this case, the female child deserves more detailed attention, whose epidemiological surveys show delays in the development of the motor system.

**Figure 3:**
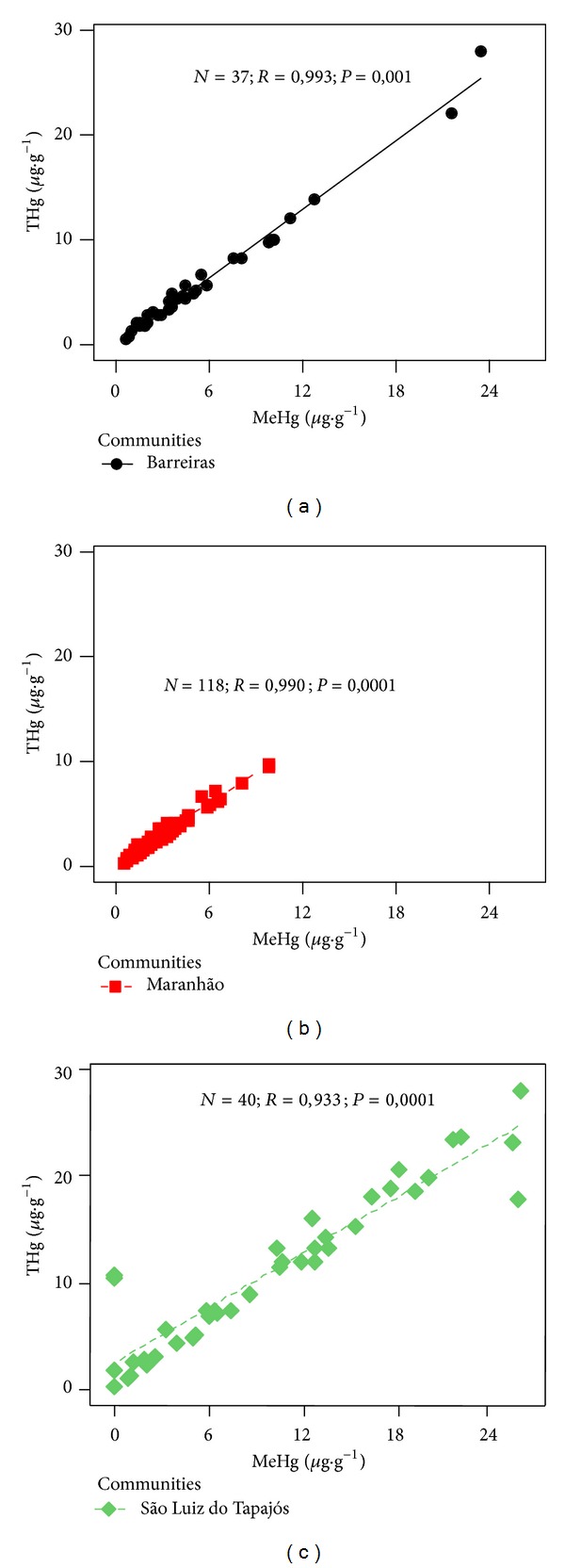
Correlation between levels of THg and MeHg in Barreiras, São Luiz do Tapajós, and Maranhão. Confidence interval is 95%.

In the analysis of correlation, a strong correlation between the levels of THg and MeHg in hair of children of Barreiras (*R* = 0.99 and *P* = 0.0001), São Luiz do Tapajós (*R* = 0.93 and *P* = 0.0001), and Maranhão (*R* = 0.99 and *P* = 0.0001), Figure 3, was observed. The strong correlation coefficient found between THg and MeHg in hair and the high percentage of MeHg (>90%) suggest that the hair can be considered as an excellent biomarker of MeHg exposure in children, especially in communities where there is the record of high fish consumption by these individuals at all stages of life [1, 24, 25]. Consequently, the measurement of Hg levels in hair is considered the main indicator of exposure in populations exposed to MeHg and is used to define international guidelines [26].

**Figure 4:**
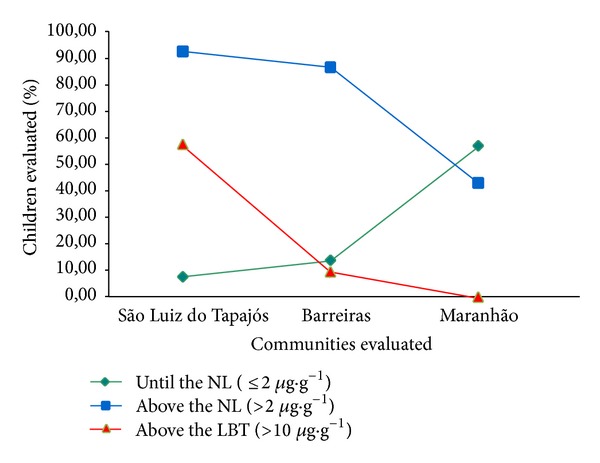
Percentage number of children with levels of THg until normality limit (NL), above the normality limit (NL) and above limits of biological tolerance (LBT).

In Barreiras, despite the fact that the found THg average levels were below the limits of biological tolerance (LBT = 10 *μ*g·g^−1^) established by World Health Organization (WHO), about 10% of children of all age groups had THg levels above this limit and 86.48% of children had shown THg levels above the normality limit (NL = 2 *μ*g·g^−1^) proposed by WHO, reaching a maximum concentration of 27.82 *μ*g·g^−1^, THg level almost three times higher than LBT. In São Luiz do Tapajós, 92.5% of children had THg levels above the NL and 57.5% reached concentrations above LBT, reaching the highest concentration of 28.17 *μ*g·g^−1^. In the Maranhão community, despite being a candidate region to control area, about 43% of children had THg levels above the NL (2 *μ*g·g^−1^), but there were no children with levels above the LBT of 10 *μ*g·g^−1^ (Figure 4). These results are strong indication that, even today, the children of the communities located on the banks of the Tapajós River are more exposed to MeHg mainly than those of the community of São Luiz do Tapajós.

**Figure 5:**
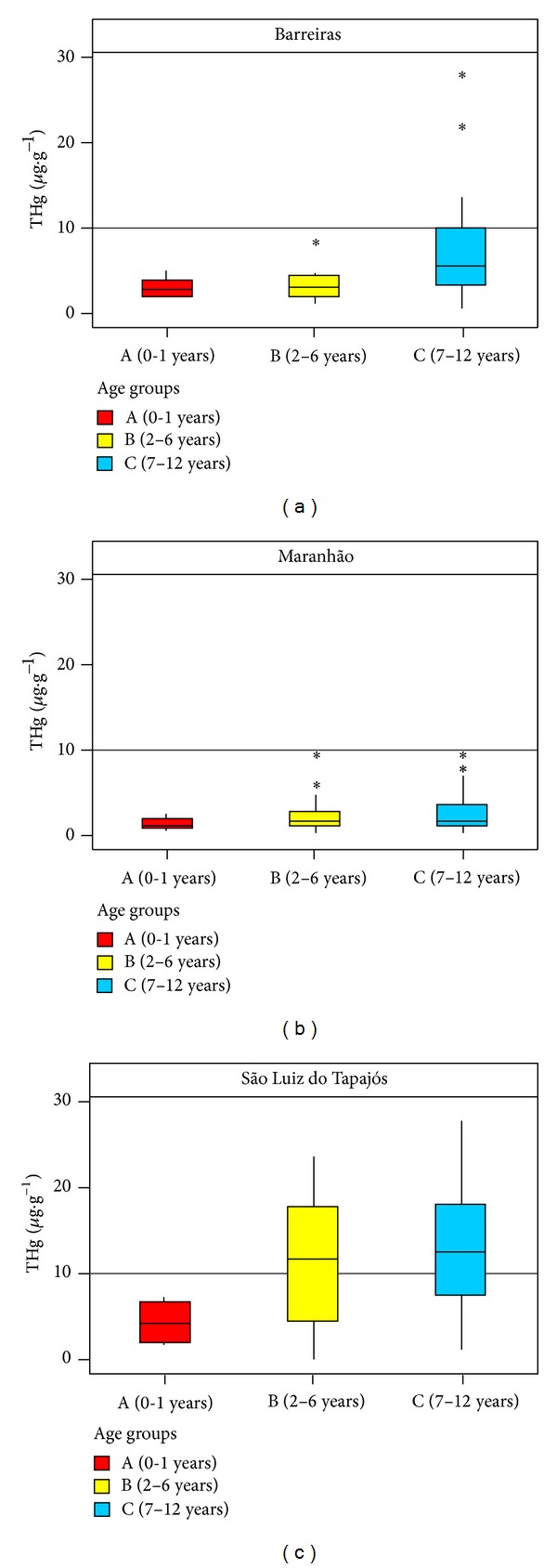
Box plot average levels of THg in hair of children by age groups. The horizontal line represents the limits of biological tolerance (LBT) by WHO (10 *μ*g·g^−1^).

The average of THg concentrations per age groups is provided in box plot in Figure 5. In Barreiras and São Luiz do Tapajós, the highest levels of THg were found in children of the C group (5.5 and 12.5 *μ*g·g^−1^, resp.); these results may be related to the time of exposure of children of this group, that is larger compared with A and B groups. In the Maranhão community, the highest levels of THg were found in children of the B group (1.7 *μ*g·g^−1^). The communities of Barreiras, São Luiz do Tapajós, and Maranhão showed different levels of mercury in different age groups, confirming previous reports that different mercury levels can be found in different age groups [18, 23, 27].

Regarding the time (in months) of breast-feeding, there was no significant correlation with MeHg in the communities of Barreiras (*R* = −0.01, *P* = 0.971) and São Luiz dos Tapajós (*R* = −0.05, *P* = 0.763). In the Maranhão community, information was not collected on duration of breast-feeding of children. This can be explained by the fact that inorganic mercury is more easily transmitted from blood to milk than the methylmercury [11, 28–30]. The preferential inorganic Hg distribution to breast milk is consistent with the association between Hg in plasma, since MeHg is preferably partitioned to erythrocytes [31]. Studies also showed that the mercury of the breastfed children's hair was not significantly correlated with levels of mercury in breast milk [32].

In the community of Barreiras, there was a significant correlation (*R* = 0.67, *P* = 0.0001) between the levels of MeHg found with the current weight of children. The consumption of food, including fish, is directly proportional to the weight of the children. Then, this may result in elevated caloric and protein intake, which can increase the levels of childhood exposure to mercury [11]. Furthermore, children may have not yet fully developed the metabolic excretion pathways, resulting in inefficiency of detoxification and physiological elimination of contaminants [11]. This result was not found for the community of São Luiz do Tapajós (*R* = −0.02, *P* = 0.892).

**Figure 6:**
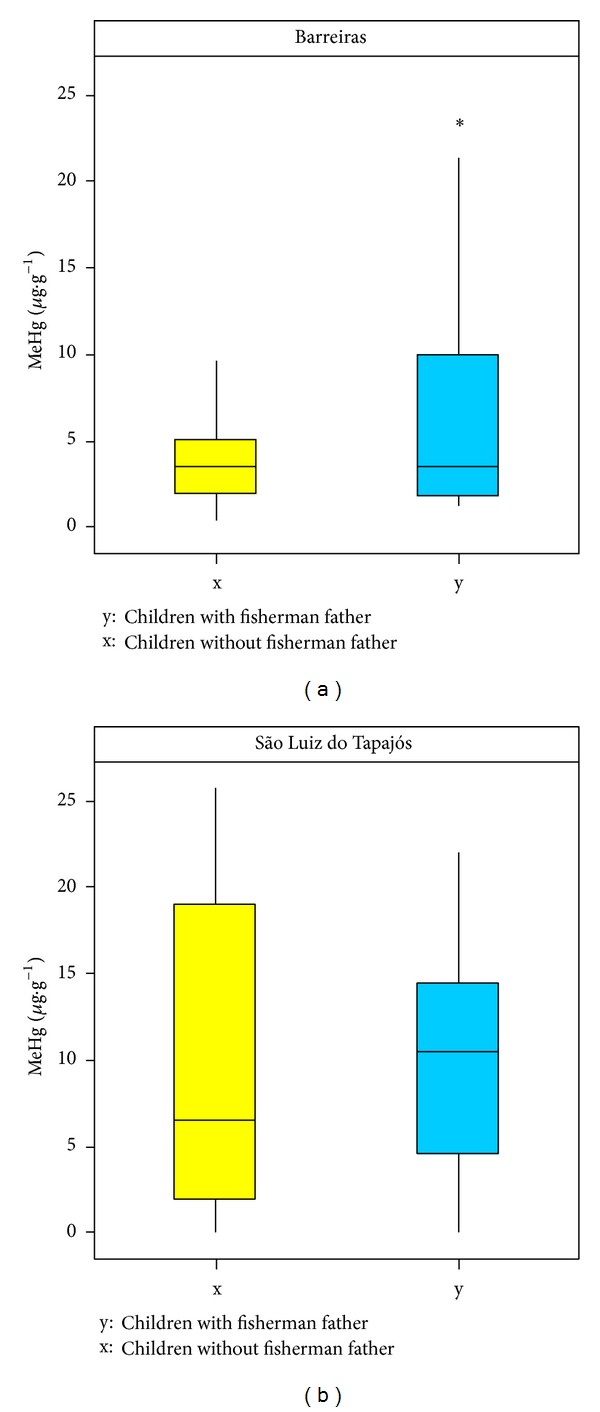
Box plot levels of MeHg in Barreiras and São Luiz do Tapajós* versus* paternal occupation.

In relation to family life, children were divided into two groups (x and y), namely, y represented children with fisherman father, and x was related to children whose fathers were not fisherman. The children of the (y) group had higher average levels of MeHg in relation to the children of (x) group in both communities (Figure 6). In the community of Barreiras, the children of (y) group showed average levels of MeHg of 7.33 *μ*g·g^−1^ (3.38–11 : 27), while, in the (x) group ones, it was 3.75 *μ*g·g^−1^ (2.68–4.81). In São Luiz do Tapajós, although the (y) group had showed higher average levels of MeHg, 10.54 *μ*g·g^−1^ (4.88–10.67), compared with the values of (x) group, 6.44 *μ*g·g^−1^ (1.73–16.11), this group had children with mercury levels above the ones established by WHO (10 *μ*g·g^−1^). Knowing that the aquatic biota is the main mercury transfer form from contaminated environment to humans in riverside communities [4, 9, 18, 27, 33], this result is justified, since it has been found, through epidemiological questionnaire, that 76.62% of the children of this region have fish as main source of dietary protein.

In studies in humans in the Tapajós River Basin, possible reduction of Hg levels in recent years [34] has been reported. In 2001, the Hg levels obtained average of 20 *μ*g·g^−1^ [1, 25, 35, 36]. However, further studies in the same populations showed lower levels, approximately 15 *μ*g·g^−1^ [5, 8, 13, 18, 22, 37], but these levels are still above LBT (10 *μ*g·g^−1^) established by WHO. In children, this tendency is also observed. In a study in the community of São Luiz do Tapajós in 1999, the THg levels found in children were 15 *μ*g·g^−1^ [36], and, in 2007, such levels were 13.39 *μ*g·g^−1^ [18]. Our results confirm a possible reduction of Hg levels in children from the communities of São Luiz do Tapajós and Barreiras (11.41 and 5.64 *μ*g·g^−1^, resp.). In São Luiz do Tapajós, despite having average levels of THg lower than those found in previous studies, this average is still above LBT.

## 4. Conclusions

Mercury exposure represents a growing health problem, mainly for riverside children, who are particularly susceptible to the effects of mercury exposure during specific periods of rapid growth and development [11]. The riverside communities of the Tapajós Valley are exposed to mercury for decades and despite the fact that recent studies show a trend of decrease of mercury levels in these populations [34], in this research these concentrations in the children hair still meet the above biological tolerance limit established by WHO (10 *μ*g·g^−1^).

The strong correlation coefficient (*R* = 0.96 and *P* = 0.0001) confirms hair as an excellent biomarker of human exposure to the organic mercury in riverside populations of the Tapajós, which has the consumption of fish daily as main feed protein source.

The results showed that the decrease in craft production in the region begins to be reflected in decreased levels of mercury in the children of the region. This decrease, although still small in scale, represents a decrease of environmental exposure to mercury in the Amazon.

Children of the Tapajós had higher average levels of mercury compared to children of the community Maranhão (area without the influence of mining). These data alerts us about the importance of comparative studies in order to obtain accurate assessments and help to establish reference levels of mercury in exposed and not exposed children in the Amazon. It also becomes relevant an clinical, epidemiological and laboratory study more refined in children from Barreiras community, who presented high levels of Hg and recorded problems in the development of the motor system.

## Figures and Tables

**Table 1:** Analytical recovery of THg and MeHg in Certificate Reference Material (CRM).

CRM	Analyte	N*	Mean ± SD (µg·g^−1^)	Reference values (µg·g^−1^)	Recovery (%)
IAEA 086	THg	38	0.568 ± 0.084	0.573	99.21
IAEA 085	MeHg	5	23.2 ± 5.35	20.73	94.35

*Number of samples.

**Table 2:** The average results of THg and MeHg and the mean percentage of MeHg.

Communities	N*	HgT (µg·g^−1^)		MeHg (µg·g^−1^)		% MeHg**
		Mean ± SD	Range	Mean ± SD	Range	
Maranhão	139	2.27 ± 1.84	0.13–9.54	2.11 ± 1.82	0.12–9.54	93.17
S.L. do Tapajós	40	11.41 ± 7.16	1.08–28.17	10.30 ± 7.67	0.96–25.74	90.27
Barreiras	37	5.64 ± 5.55	0.43–27.82	5.20 ± 5.06	0.43–23.20	92.20

*Number of samples

**Mean percentage of methylmercury (MeHg/THg × 100).
